# Cerebrovascular reactivity after functional activation of the Brain using Photic Stimulation in Migraine and Tension Type Headache: a transcranial doppler Ultrasonography Study

**DOI:** 10.1186/s12883-023-03153-2

**Published:** 2023-03-17

**Authors:** Eman M Khedr, Mohammed A Abbas, Ayman Gamea, Mohamed A Sadek, Ahmed F Zaki

**Affiliations:** 1 Department of Neuropsychiatry, Faculty of Medicine, Assiut University Hospital, Assiut, Egypt; 2 Neuropsychiatric Department, Faculty of Medicine, Aswan University Hospital, Aswan, Egypt; 3 Neuropsychiatry department Faculty of Medicine, Luxor University, Luxor, Egypt; 4 Neuropsychiatric Department, Faculty of Medicine, South Valley University, Qena University Hospital, Qena, Egypt

**Keywords:** Migraine, Tension type headache, Cerebrovascular reactivity, Transcranial Doppler Sonography; cerebral blood flow velocities

## Abstract

**Background:**

Previous studies in headache patients measured the cerebrovascular reactivity (CVR) in response to photic stimulation but they have yielded contradictory results. The purpose of study was to measure CVR of both migraine and chronic tension headache (TTH) patients in response to photic stimulation.

**Methods:**

The study included 37 migraineurs and 24 chronic TTH patients compared with 50 age- and sex-matched healthy volunteers. Peak systolic, end diastolic, mean flow velocities and CVR (PSV, EDV, MFV, and CVR) were measured using TCD ultrasonography of the middle, anterior, posterior cerebral and vertebral arteries (MCA, ACA, PCA, and VA) before and after 100 s of 14 Hz photic stimulation.

**Results:**

A three-way repeated measures ANOVA interaction with main factors of Vessels (MCA, ACA, PCA, VA), Time (pre-post photic) and Groups (migraine, TTH, and control group) revealed significant 3-way interactions for measures of PSV (P = 0.012) and MFV (*P = 0.043*). In the migraine patients there was significantly higher PSV, EDV, and MFV in the MCA, ACA, and PCA after photic stimulation compared with baseline. The CVR of the MCA was also significantly higher in migraineurs than controls. In the TTH group, there was significantly higher PSV, EDV, and MFV (P = 0.003, 0.012, 0.002 respectively) in the VA after photic stimulation than at baseline. The CVR was significantly higher in the VA of TTH patients than controls.

**Conclusion:**

Compared with controls after photic stimulation, the higher CVR of the MCA in migraineurs and of the VA in TTH patients could be used as diagnostic tool to differentiate between the two types of headaches.

## Background

One of the pathophysiological alterations in migraine is neuronal hypersensitivity to various intrinsic and extrinsic stimuli. This can be detected by changes in cerebral vasoreactivity [1] that can be measured using transcranial doppler (TCD) ultrasonography. One commonly used stimulus is hypercapnia, with a recent meta-analysis showing that the cerebrovascular reactivity (CVR) to hypercapnia is lower in the posterior circulation of migraineurs, particularly those without aura [2]. Others have used visual stimulation. Zaletel et al. [3] used a checkerboard stimulus and found that visually evoked cerebral blood flow velocity responses (VEFR) were higher interictally in 30 migraineurs than in a control group. Backer et al. [4] found a steady increase in the cerebral blood flow velocity (CBFV) of migraineurs while it habituated in controls. Sedighi et al. [5] examined the effect on blood flow of a flickering light stimulus lasting 100 s on patients with migraine and a control group. They found that at baseline, there was a higher peak systolic velocity in the posterior cerebral artery of migraineurs than the control group, while peak systolic velocity during stimulation was not statistically significant. Nedeltchev et al. [6] assessed CBFV changes in MCA and PCA in relation to repetitive checkerboard visual stimulation in19 migraineurs and 19 normal volunteers. During visual stimulation the CBFV was significantly larger and steeper in migraineurs than in controls.

In population-based studies, 78% of headache patients are diagnosed with tension type headache (TTH). The clinical diagnosis is based chiefly on negative features (absence of symptoms that characterize other primary or secondary headaches). However, a minority of TTH patients can have some of these features. For example, 18% have pulsatile headache, 10% unilateral pain, 28% aggravation on routine physical activity, 18% anorexia, 4% nausea, and 11% photophobia [7]. Simultaneous recording of the CVR of the middle cerebral artery (MCA) to visual stimulation revealed an increase of cerebral blood flow velocity of over 10 cml/s in 6/15 children with migraine and TTH [8]. Yet CVR is rarely measured in TTH despite the presence of photophobia in some cases. So, the question we ask in the present study is whether their CBF response differs from that of patients with migraine. There have been few studies of this in the past [3–6, 8–10] and none of these references say anything definite about TTH. The aim of this study was to compare interictal CVR in patients with migraine and TTH in response to photic stimulation.

## Methods

Sixty-one patients with headache were recruited consecutively from the outpatient’s clinic of Qena University Hospital during the period from 1st January 2019 to 31th December 2019). 37 had migraine and 24 had TTH according to criteria of the International Society of Headache [10].

Inclusion criteria: both sexes were included with an age range from 20 to 50 years and at least a 6-month history of headache. Exclusion criteria: patients with hypertension, diabetes mellitus or severe medical disease, vascular disease, stroke, addiction disorder, or patients under treatment with calcium or beta blockers as well as alcohol abuse. None had previously received Botox injections or calcitonin gene-related peptide (CGRP) inhibitors. Patients receiving triptans or ergot drugs as urgent treatment to abort an attack during the study were excluded as were patients taking any antidepressants for at least 3 days before the examination. (See inclusion and exclusion criteria in Khedr et al. 2022).

We classified each type of headache into two subgroups according to the International Society of Headache: episodic; <15 attacks per month and chronic; ≥ 15 attacks of headache/month (16 chronic and 8 episodic TTH and 23 episodic and 14 chronic migraine)[11]. However as there was no significant difference between episodic and chronic in each type of headache or between migraine with aura (16 cases) or without aura (21 cases) in TCD data at rest as reported in the our previous paper (Khedr et al. (2022)[12], we combined all patients with migraine together (37 cases) versus all patients with TTH (24 cases).

The data were compared with 50 control age- and sex-matched healthy volunteers with the same exclusion criteria. A full neurological history and neurological examination was obtained in all the patients. A Computerized brain tomography of brain was done for each patient to exclude secondary headache. All patients were headache-free for at least 3 days at the time of the examination.

### Transcranial doppler ultrasonography examination

The TCD examination was performed in a quiet room according to previously recommended practice standards using a SAMSUNG HS60 DEVICE manufactured in South Korea with a faced array probe 2–4 Hz. Peak systolic velocity (PSV), end diastolic velocity (EDV), mean flow velocity (MFV) and pulsatile index (PI) were obtained for the right side of the head for middle cerebral artery (MCA) at depth 40–65 mm, anterior cerebral artery (ACA) at 60–75 mm, posterior cerebral artery (PCA) at 55–75 mm, and vertebral artery (VA) at 40–75 mm. The MCA, and ACA were defined as anterior circulation, while the VA, and PCA were classified as posterior circulation. The mean flow velocity was calculated as EDV plus one-third of the difference between PSV and EDV (i.e. [2xEDV + PSV] / 3). Because asymmetric CBFVs were not related with the headache side or migraine with/without aura [13]. we select the right side of the head For TCD study in all patients and controls. The pulsatility index (PI) was used as a measure of stiffness of a blood vessel; a higher PI correlates with a stiffer blood vessel. PI is calculated by subtracting EDV from PSV and dividing the value by MFV [14].

During insonation, participants were given 14 Hz photic flash stimulation for 100 s from a distance of approximately 1 m. The flow velocity was recorded before and 50 s after the end of stimulation at the same depth and site [4].

**The cerebrovascular reactivity (CVR)** was calculated as Vstim − Vrest / Vrest*100 where Vstim is the mean blood flow velocity during the stimulation, and Vrest is the baseline mean flow velocity during the initial 5 min prior to stimulation [10].

### Consent and ethical approval

Written confirmed consent was obtained from each participant after explaining all point of the study, and local ethical committee of Faculty of Medicine South Valley University approved the study.

### Statistical analysis

All data were analyzed with the aid of the SPSS ver.16. Descriptive statistics, cross- tabs and frequency tables were used to describe some of the basic variables. Most of the data were normally distributed as checked by Shapiro-Wilk test except some minor deviations from normality in some parameters, but given the robustness of ANOVA, the authors decided to use this throughout the analysis. T-test was performed to compare continuous variables between groups which are expressed as mean ± SD data. Categorical variables were compared by Fisher’s exact 2-tailed test or by the Chi Square test. Three Ways-ANOVA was performed to detect interaction between groups (4 vessels (MCA, ACA, PCA, VA) X Time (pre and post photic) X 3 groups (Migraine, tension headache and controls).

Two Way-ANOVA repeated measurements analysis for each vessel separately with the main effect of time “Pre and post stimulation “X groups (three groups) was used to compare the differential effects of the photic stimulation on each TCD parameters scores for each vessel. When necessary, a Greenhouse–Geisser correction was applied to correct for non-sphericity.

Post hoc T-test was used to compare pre versus post photic stimulations for all Doppler parameters for each vessel. Kruskal-Wallis H was performed to measure the significant between the three groups in CVR. Pearson’s coefficient correlation was performed between CVR data of each artery with different clinical parameters. The accepted significance threshold was p < 0.05.

## Results

Table 1*showed* Demographic and Clinical Data of studied groups. There were no significant differences between groups in mean age, and sex distribution between studied groups. There was no significant difference between patient groups in duration of illness. The frequency of attacks was significantly higher in tension headache group and family history was significantly higher among migraine than other groups. Because there were no significant differences between migraine with aura and migraine without aura in different parameters of TCD we combined them into one group. Out of 37 patients with migraine 16 patients had aura, 10 of whom experienced visual symptoms of zigzags and flashes of lights. The remaining 6 patients had a sensory aura in the form of mouth numbness and dizziness. Visual stimuli as a trigger for headache were reported 5 patients who had migraine and aura, 5 patients who had migraine without aura and in 1 patient with TTH.

**Table 1:** Demographic and Clinical Data of studied groups

Variables/Group		Migraine (37 cases)	Tension headache (24 cases)	Control (50 normal)	P value between different groups
Age (mean ± SD)		30.54 ± 8.3	35.5 ± 7.4	31.4 ± 8.3	0.119 (between 3 groups)
**Sex**	Male/female	18/19	8/16	27/23	0.097 (between 3 groups)
**Duration of disease in months (mean** **±** **SD)**		33.22 ± 22.5	33.7 ± 33.8	-	0.981 (between Tension and migraine group)
**Frequency of attacks/month (mean** **±** **SD)**		12.11 ± 4.47	20.33 ± 4.5	-	0.001(between Tension and migraine group)
**Family history**		24(81.25%)	5(20.8%)	-	0.001(between Tension and migraine group)

Three Way-ANOVA repeated measure analysis revealed a significant Vessels (MCA, ACA, PCA, VA) X Time (pre and post photic) X Group (Migraine, tension headache and controls) interaction for PSV (P = 0.012, f = 3.8, df = 6(2.7)), and the MFV revealed a significant interaction (*Vessels X time X groups) with P = 0.043*, f = 2.8, df = 6 (2.86)), with no significant interaction neither for EDV (Vessels X time X groups with P = 0.231, f = 1.44, df = 3.15, nor for PI Vessels X time X groups (P = 0.35, f = 1.1, df = 6(2.5)).

Table 2 shows the changes in MCA blood flow pre and post photic stimulations. A two-way repeated measures ANOVA showed a significant interaction of Time X Groups for PSV and MFV (P = 0.001, and 0.043 respectively). Post hoc T-tests (pre versus post photic stimulation) for each group separately showed a significantly higher PSV, EDV, and MFV after photic stimulation (P = < 0.001, 0.008, and 0.001 respectively) in the migraine group while no such changes were seen in the tension headache and control groups.

**Table 2:** Duplex parameters of middle cerebral artery (MCA) before and after photic stimulation among studied groups

Duplex parameters			Migraine Headache (37 cases)	Tension headache (24 cases)	Control (50 normal)	Two way ANOVA Time (before versus after stimulation)X groups (three groups)
**PSV**	**Before**		83.54 ± 23.3	68.9 ± 17.3	88.1 ± 21.7	**P = 0.001** F = 7.9 Df = 2
	**After**		93.4 ± 24.02	71.6 ± 14.8	89.8 ± 20.9	
	**Before vs. after**	**T-value**	6.89	0.855	1.7	
		**P value**	**0.001**	0.402	0.082	
**EDV**	**Before**		37.6 ± 11.83	31.8 ± 10.5	35.8 ± 11.4	P = 0.145 F = 1.9 Df :2
	**After**		41.5 ± 11.13	32.76 ± 8.56	36.7 ± 10.6	
	**Before vs. after**	**T value**	2.83	0.439	1.06	
		**P value**	**0.008**	0.665	0.293	
**Mean flow velocity**	**Before**		52.94 ± 15.3	44.2 ± 12.4	53.1 ± 13.8	**P = 0.04** F = 3.2 Df = 2
	**After**		58.8 ± 14.45	45.7 ± 10.2	55.1 ± 13.6	
	**Before vs. after**	**T value**	5.03	0.638	1. 9	
		**P value**	**0.001**	0.530	0.06	
**PI**	**Before**		0.86 ± 0.15	0.94 ± 0.87	0.89 ± 0.10	P = 0.42 F = 0.85 Df = 2
	**After**		0.83 ± 0.15	0.79 ± 0.13	0.88 ± 0.13	
	**Before vs. after**	**T-value**	0.75	0.794	0.349	
		**P value**	0.45	0.436	0.728	

PSV; Peak Systolic velocity, EDV; End Diastolic Velocity, PI; pulsatility Index, Mean; mean flow velocity, P value is significant < 0.05

Table 3 shows the changes in ACA blood flow pre and post photic stimulations. There was no Time X Groups interaction for any TCD parameter. Exploratory analysis of the effect of photic stimulation in each group separately showed that there was a significantly higher, EDV, MFV and PI in the ACA after photic stimulation only in the migraine group (P = 0.001, 0.001, and 0.019, respectively) but there were no such changes in the other groups.

**Table 3:** Duplex parameters of anterior cerebral artery (ACA) before and after photic stimulation among studied groups

Duplex parameters			Migraine Headache (37 cases)	Tension headache (24 cases)	Control 50 normal	Two way ANOVA Time (before versus after stimulation)X groups (three groups)
**PSV**	**Before mean** **±** **SD**		61.5 ± 14.18	60.4 ± 13.8	59.3 ± 13.5	P = 0.44 F = 0.82 Df = 2
	**After mean** **±** **SD**		63.9 ± 14.84	63.3 ± 14.03	60.1 ± 13.7	
	**Before vs. after**	**T value**	1.77	1.419	1.125	
		**P value**	0.08	0.169	0.266	
**EDV**	**Before mean** **±** **SD**		25.8 ± 6.2	27.2 ± 8.5	23.4 ± 6.4	P = 0.076 F = 2.6 Df = 2
	**After mean** **±** **SD**		29.18 ± 7.5	27.4 ± 8.4	24.5 ± 6.5	
	**Before vs. after**	**T value**	3.68	0.270	1.6	
		**P value**	**0.001**	0.911	0.111	
**Mean**	**Before mean** **±** **SD**		37.74 ± 7.9	38.3 ± 9.5	35.4 ± 8.05	P = 0.21 F = 1.6 Df = 2
	**After mean** **±** **SD**		40.7 ± 9.1	39.3 ± 9.8	36.6 ± 8.1	
	**Before vs. after**	**T value**	3.48	0.520	1.9	
		**P value**	**0.001**	0.487	0.06	
**PI**	**Before mean** **±** **SD**		0.87 ± 0.19	0.9 ± 0.21	0.91 ± 0.14	P = 0.32 F = 1.13 Df = 2
	**After mean** **±** **SD**		0.76 ± 0.15	0.86 ± 0.16	0.86 ± 0.17	
	**Before vs. after**	**T value**	2.4	1.053	1.8	
		**P value**	**0.019**	0.303	0.072	

PSV; Peak Systolic velocity, EDV; End Diastolic Velocity, PI; pulsatility Index, Mean; mean flow velocity, P value is significant < 0.05

Table 4 shows the changes in PCA blood flow. There was a significant Time X Group interaction for PSV (P = 0.043) only. Exploratory post hoc analysis of each group separately showed that there was a significantly higher PSV, EDV, and MFV of the PCA after compared with before photic stimulation in the migraine group (P = < 0.0001for each) while there were no such changes in the TTH and control groups.

**Table 4:** The Duplex parameters of posterior cerebral artery (PCA) before and after photic stimulation among studied groups

Duplex parameters			Migraine Headache (37 cases)	Tension headache (24 cases)	Control 50 normal	Two way ANOVA Time (before versus after stimulation)X groups (three groups)
**PSV**	**Before mean** **±** **SD**		57.3 ± 10.2	50.4 ± 12.9	55.8 ± 14.1	**P = 0.043** F:=3.2 Df = 2
	**After mean** **±** **SD**		61.7 ± 10.65	52.6 ± 15.1	56.4 ± 14.5	
	**Before vs. after**	**T value**	4.22	1.011	0.796	
		**P value**	**0.001**	0.322	0.430	
**EDV**	**Before mean** **±** **SD**		26.6 ± 6.9	23.3 ± 7.18	24.4 ± 7.9	P = 0.084 F = 2.5 Df :2
	**After mean** **±** **SD**		29.69 ± 7.5	23.9 ± 7.2	25.1 ± 9	
	**Before vs. after**	**T value**	4.2	0.431	0.93	
		**P value**	**0.001**	0.671	0.357	
**Mean**	**Before mean** **±** **SD**		37.01 ± 7.8	32.3 ± 8.9	34.5 ± 9.5	P = 0.218 F = 1. 5 Df = 2
	**After mean** **±** **SD**		40.38 ± 8.1	33.5 ± 9.7	35.8 ± 10.4	
	**Before vs. after**	**T-value**	4.8	0.703	1.8	
		**P value**	**0.001**	0.489	0.07	
**PI**	**Before mean** **±** **SD**		0.8 ± 0.14	0.81 ± 0.22	0.84 ± 0.13	P = 0.69 F = 0.364 Df = 2
	**After mean** **±** **SD**		0.77 ± 0.18	0.79 ± 0.14	0.84 ± 0.17	
	**Before vs. after**	**T-value**	1.4	0.402	0.124	
		**P value**	0.168	0.691	0.902	

PSV; Peak Systolic velocity, EDV; End Diastolic Velocity, PI; pulsatility Index, Mean; mean flow velocity, P value is significant < 0.05

Table 5 shows the changes in VA blood flow. There was no Time X Group interaction for any TCD parameter. Exploratory analysis in each group separately showed a significantly higher PSV, EDV, and MFV (P = 0.003, 0.012, 0.002 respectively) in the VA after photic stimulation than before stimulation in the tension headache group only, with no changes observed in the other groups except a weak significant PSV in migraine group after photic stimulation compared to before stimulation (P = 0.04).

**Table 5:** Duplex parameters of vertebral artery (VA) before and after photic stimulation among studied group

Duplex parameters			Migraine headache (37 cases)	Tension headache (24 cases)	Control 50 normal	Two way ANOVA Time (before versus after stimulation)X groups (three groups)
**PSV**	**Before mean** **±** **SD**		50.3 ± 13.1	46.1 ± 12.8	49.7 ± 10.9	P = 0.062 F = 2.8 Df = 2
	**After mean** **±** **SD**		53.4 ± 12.3	51.6 ± 16.6	50.8 ± 11.6	
	**Before vs. after**	**T value**	2.06	3.262	1.6	
		**P value**	**0.04**	**0.003**	0.11	
**EDV**	**Before mean** **±** **SD**		22.2 ± 5.8	19.4 ± 4.9	20.2 ± 6.2	**P = 0.102** F = 2.3 Df = 2
	**After mean** **±** **SD**		22.9 ± 7.04	22.2 ± 7.8	20.5 ± 6.9	
	**Before vs. after**	**T value**	0.81	2.725	0.444	
		**P value**	0.41	**0.012**	0.659	
**Mean**	**Before mean** **±** **SD**		31.9 ± 7.5	28.3 ± 6.7	30.4 ± 7.2	P = 0.07 F: 2.7 Df = 2
	**After mean** **±** **SD**		33.1 ± 7.8	32.0 ± 9.3	30.7 ± 7.9	
	**Before vs. after**	**T value**	1.15	3.405	1.3	
		**P value**	0.25	**0.002**	0.197	
**PI**	**Before mean** **±** **SD**		0.8 ± 0.18	0.86 ± 0.24	1.3 ± 1.9	P = 0.26 F: 1.36 Df = 2
	**After mean** **±** **SD**		0.9 ± 0.25	0.93 ± 0.35	0.93 ± 0.22	
	**Before vs. after**	**T value**	0.24	1.169	1.39	
		**P value**	0.8	0.255	0.171	

PSV; Peak Systolic velocity, EDV; End Diastolic Velocity, PI; pulsatility Index, Mean; mean flow velocity, P value is significant < 0.05

Table 6 shows the cerebrovascular reactivity after photic stimulation in the three groups (CVR was around 15% in migraine patients while in the controls the change was only 5% particularly for MCA). Kruskal-Wallace tests between the three groups showed a significant difference only for CVR in the MCA (0.006). Comparing each patient group with the control group using a Mann-Whitney test showed a significantly higher CVR in the MCA of the migraine group and a higher CVR in the VA of the TTH group compared with controls (P = < 0.0001, 0.022 respectively) as well as between the TTH and migraine groups (0.015) in the MCA.

**Table 6:** Cerebrovascular reactivity (% changes) in response to photic stimulation in the three groups

CVR of different vessles	Migraine (37cases) (% change)	Tension headache (24 cases) (% change)	Control (50 normal) (% change)	P- value between control vs. tension headache	P- value between control vs. migraine	P- value between the three groups Kruskal-Wallis
CVR of MCA	15.0%	8.7%	5.0%	0.47	0.012	0.013
CVR of ACA	12.0%	4.5%	7.3%	0.45	0.16	0.263
CVR of PCA	10.8%	6.4%	6.1%	0.95	0.33	0.267
CVR of VA	8.4%	13.2%	3.3%	0.014	0.33	0.130

CVR; cerebrovascular reactivity, MCA; middle **cerebral artery**, ACA; anterior cerebral artery

PCR; **posterior cerebral artery**, VA; **vertebral artery**

**Fig. 1:**
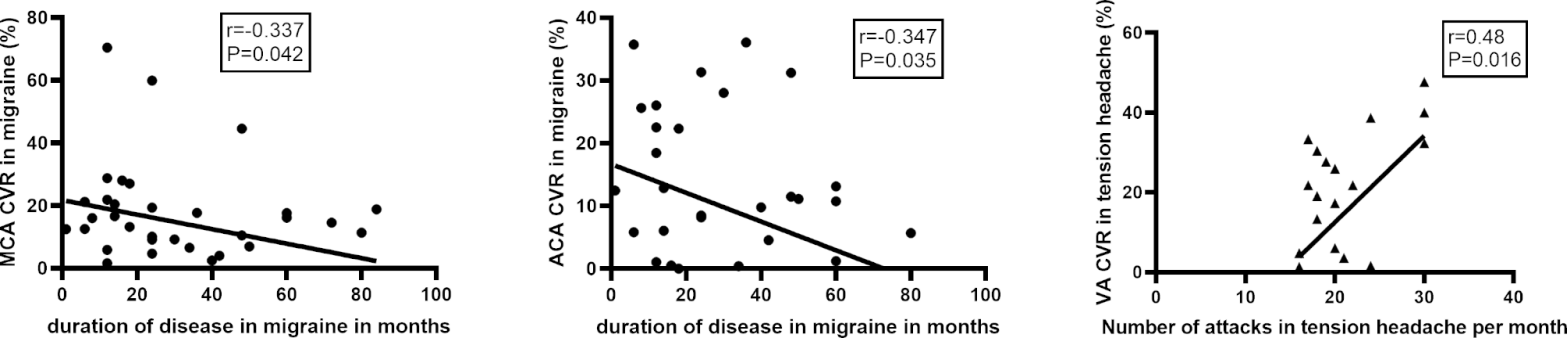
Correlation between CVR of MCA and ACA of migraine patients and duration of disease in months (1st and 2nd trace), and correlation between CVR of VA and number of attacks per month in chronic tension headache (3rd trace). There are significant negative correlation between CVR of MCA and ACA in migraineurs patients and duration of illness (P = 0.042, and 0.035 respectively). A significant positive correlation between CVR of VA in TTH group and frequency of attacks (P = 0.016)

Table 7**and** Fig. 1 show the correlation between the CVR of the different arteries and demographic and clinical data: There were significant negative correlations between duration of illness and both CVR of MCA and ACA in migraineurs (P = 0.042, and 0.035 respectively). There was a significant positive correlation between frequency of attacks and CVR in the VA of the TTH group (P = 0.018), while other clinical parameters had no significant correlations with CVR.

**Table 7:** Correlation between CVR of the four vessels and demographic and clinical data of studied group

		Migraineurs groups				Tension Headache group			
		CVR of MCA	CVR of ACA	CVR of PCA	CVR of VA	CVR of MCA	CVR of ACA	CVR of PCA	CVR of VA
Age	Correlation Coefficient	-0.169	-0.091	0.053	0.074	0.06	-0.21	-0.05	0.1
	P value	0.318	0.594	0.754	0.664	0.76	0.3	0.79	0.61
Body Mass Index (BMI)	Correlation Coefficient	-0.160	0.128	0.101	0.059	0.05	0.14	-0.21	0.17
	P value	0.345	0.452	0.552	0.727	0.8	0.5	0.3	0.4
Duration of the disease	Correlation Coefficient	**-0.337**	-0.347*	0.304	0.153	-0.04	0.06	-0.04	0.27
	P value	**0.042**	**0.035**	0.067	0.366	0.8	0.75	0.82	0.18
Number of attacks	Correlation Coefficient	0.107	-0.102	0.113	-0.105	0.2	-0.03	0.23	**0.48**
	P value	0.527	0.549	0.505	0.538	0.33	0.86	0.27	**0.016**

CVR; cerebrovascular reactivity, MCA; middle cerebral artery, ACA; anterior cerebral artery

PCR; posterior cerebral artery, VA; vertebral artery

## Discussion

There are still some doubts about how different stimuli in the interictal phase change blood flow velocity and cerebral vasomotor reactivity in the intracranial arteries of migraineurs and patients with TTH. The current study was performed to determine the changes after photic stimulation. One of the main findings was a significant increase of the MFV of the MCA, ACA and PCA after photic stimulation in migraineurs which differed from that in the control group in the MCA. There were no significant changes in the TTH or control group after photic stimulation. The implication is that there is a stronger CVR in the MCA of migraineurs than in controls (CVR was around 15% in migraine patients while in the controls the change was only 5% for MCA). The altered cerebrovascular response in migraineurs could be due to altered perceptual processing or an imbalanced reaction of the blood vessel diameter to the vasoactive stimulus which is thought to play a role in triggering migraine attacks. Another possible explanation is the loss of habituation to photic stimulation in migraine. Thie et al. [10] also found that the increase of MFV in the MCA during a cognitive task and photic stimulation was greater in migraineurs than in controls (9.1% in migraineurs vs. 5.0% in control; P = 0.06 for the cognitive task, and 17.4% vs. 9.9% for photic stimulation).

Min et al. [2011] applied photic stimuli to migraineurs taking propranolol for prophylaxis and found that the CVR in the PCA was decreased but the baseline MFV was unchanged prior to visual stimulation. They assumed that the protective effect of propranolol was not due to a direct effect on the cerebral vessels but could be due to modulation of the CVR to sensory stimulation[15]. Evidence from controlled studies recorded that neither propranolol nor flunarizine treatment influences the cerebral blood flow velocities (CBFVs) of basal cerebral arteries in migraine patients[15–17]. In the present study patients under treatment with calcium or beta blockers or CGRP, triptans or ergot drugs were excluded to eliminate these drug effects.

Consistent with our results Nedeltchev et al. [6] mentioned that the cerebrovascular responses to visual stimuli in both PCA and MCA were significantly higher in migraine patients than in controls. They considered two possible explanations. First, that autonomous hyperactivity may be more pronounced in migraineurs, i.e. systemic factors such as blood pressure, heart rate, PCO2, intracranial pressure, etc. could have caused a velocity increase in both arteries, or second, that there may be a higher level of cortical arousal in migraine [6]. One of the earliest studies in this field was Backer et al. [4] who reported a significant increase in the MCA blood flow that was maintained until 10 s after the light was off. This was not the case in the control group, who habituated rapidly to the light stimulus.

Other studies with similar results to ours include that of Sedighi et al. [5] who found a significantly increased peak systolic velocity post photic stimulation. In another study Nowak et al. [8] recorded that cerebral blood flow velocities increased over 10 cm/s in response to visual stimulation in 6 patients (2 with migraine with aura, 2 with hemicrania epileptica, 1 with migraine without aura, 1 with chronicTTH). Sedighi et al. [5] mentioned that the change of PSV in the PCA was significantly greater in the migraine group compared with the control group after visual stimulation.

The second main finding in the present study was the higher PSV, EDV, and MFV in the VA after photic stimulation in TTH than other groups, with a flow velocity over 15 cm/s, a finding that supports the possible involvement of vascular mechanisms in TTH. There was a significantly higher CVR in the VA of TTH group versus the control group (P = 0.0I22). These vascular changes of TTH may support the theory of disturbed central motor activity control of pericranial musculature in TTH sufferers. This theory has been emphasized by Olesen et al. [11] and Wallasch et al. [18], as they described vascular-supraspinal-moyogenic model of primary headaches.

The negative correlation between the CVR of both MCA and ACA and the duration migraine in our study may be due to the fact that chronic sympathetic hyper-responsiveness might decrease over time [19]. This is in agreement with Savrun et al. who suggested that analgesic overuse a long period results in a functional disorder of neuronal receptor and neurovascular reflexes and may cause a reduction of intracerebral vessel tone, leading to vasodilatation [20].

The positive correlation between the CVR of VA with the number of attacks in TTH patient that recorded in the current study, may be explained as the more frequent the attack the more disturbed central motor activity controlling the pericranial musculature [11].

## Conclusion

High PSV, EDV, MFV and CVR in response to photic stimulation predominantly in the anterior circulation were particularly evident in migraineurs. High PSV, EDV, MFV and CVR in response to photic stimulation in the VA were evident in TTH. Additionally, a complex relationship may exist between altered CVR, with the frequency and duration of illness underlies the pathophysiology of migraine and TTH respectively.

### The strengths of the study

The strengths of the present study are as follows: (1) TCD was performed for all patients and controls by a single investigator thus avoiding inter-assessor differences; (2) TCD was performed for all vessels of the anterior and posterior circulation. (3) A possible bias introduced by medication effects was excluded. The correlation between CVR (% changes) of the four vessels after photic stimulation with the demographic and clinical data provides an insight into the pathophysiology of both types of primary headache.

### Limitations and recommendation of the study

The small sample size is one of the limitations of this study. Another limitation is the unequal number of males and females in the control group in contrast to the headache groups. Another important limitation is the significant difference between the groups in headache frequency. Correlations of % change in headache days (frequency of headache) and % change in CBFVs were evaluated by Mi et al. 2017 who found positive correlations with CBFVs of MCAs in migraine patients[13]. The decision to combine data from episodic and chronic headache is another limitation as changes in the frequency of the attacks are most typically recognized as a marker of clinical types of primary headache [21–23] and the neurophysiological changes associated with frequency have rarely been investigated. We also did not compare data between the two sides of the brain to test for differences between the side with migraine pain and the side without. More extensive studies with a large sample size are needed to establish the possible vascular pathophysiology of migraine and TTH.

## Data Availability

The datasets used and/or analyzed during the current study are available from the corresponding author reasonable on request.

## Abbreviations

ACA: Anterior cerebral artery
CBF: Cerebral blood flow
CBFV: Cerebral blood flow velocity
CVR: Cerebrovascular reactivity
EDV: End diastolic velocity
MCA: Middle cerebral artery
MFV: Mean flow velocity
PCA: Posterior cerebral artery
PSV: Peak systolic velocity
TCD: Transcranial doppler
TTH: Tension Type-headache
VA: vertebral artery
VEFR: Visually evoked cerebral blood flow velocity response.

## References

[1] Carod-Artal FJ (2014). Tackling chronic migraine: current perspectives. J pain Res.

[2] Dzator JS, Howe PR, Wong RH (2021). Profiling cerebrovascular function in migraine: a systematic review and meta-analysis. J Cereb Blood Flow Metabolism.

[3] Zaletel M, Strucl M, Bajrovi F, Pogacnik T (2005). Coupling between visual evoked cerebral blood flow velocity responses and visual evoked potentials in migraneurs. Cephalalgia.

[4] Bäcker M, Sander D, Hammes M, Funke D, Deppe M, Conrad B, Tölle T (2001). Altered cerebrovascular response pattern in interictal migraine during visual stimulation. Cephalalgia.

[5] Sedighi B, Ebrahimi HA, Jabbarpour S, Shafiee K (2011). Transcranial doppler sonography diagnostic value for the cerebral flow velocity changes in the interictal phase of classic migraine. Caspian J Intern Med.

[6] Nedeltchev K, Arnold M, Schwerzmann M, Nirkko A, Lagger F, Mattle H, Sturzenegger M (2004). Cerebrovascular response to repetitive visual stimulation in interictal migraine with aura. Cephalalgia.

[7] Langemark M, Olesen J, Poulsen DL, Bech P (1988). Clinical characterization of patients with chronic tension headache. Headache.

[8] Nowak A, Gergont A, Steczkowska M. Ocena przepływu mózgowego po stymulacji świetlnej u dzieci z migreną i przewlekłymi bólami głowy typu napięciowego-wyniki wstępne [Assessment of cerebral blood flow after visual stimulation in children with a migraine and chronic tension-type headache–preliminary reports].Przegląd Lekarski2008, 65(11). 19205360

[9] Biedroń A, Kaciński M. Wpływ bodźca wzrokowego na przepływ mózgowy i wzrokowe potencjały wywołane u dzieci z migreną z aurą wzrokową[Effect of a visual stimulus on cerebral flow and visual evoked potentials in children with migraine with a visual aura].Przegląd Lekarski2010, 67(9). 21387805

[10] Thie A, Carvajal-Lizano M, Schlichting U, Spitzer K, Kunze K (1992). Multimodal tests of cerebrovascular reactivity in migraine: a transcranial Doppler study. J Neurol.

[11] Olesen J (1991). Clinical and pathophysiological observations in migraine and tension-type headache explained by integration of vascular, supraspinal and myofascial inputs. Pain.

[12] Khedr EM, Abbas MA, Gamea A, Sadek MA, Zaki AF (2022). Cerebrovascular function in tension-type headache and migraine with or without aura: Transcranial Doppler study. Sci Rep.

[13] Lee MJ, Chu MK, Choi H, Choi HA, Lee C, Chung CS (2017). Longitudinal changes in cerebral blood flow velocities in different clinical courses of migraine. Cephalalgia.

[14] Bathala L, Mehndiratta MM, Sharma VK (2013). Transcranial doppler: technique and common findings (part 1). Ann Indian Acad Neurol.

[15] Min JH, Kwon HM, Nam H (2011). The effect of propranolol on cerebrovascular reactivity to visual stimulation in migraine. J Neurol Sci.

[16] Diener HC, Peters C, Rudzio M, Noe A, Dichgans J, Haux R, Ehrmann R, Tfelt-Hansen P (1991). Ergotamine, flunarizine and sumatriptan do not change cerebral blood flow velocity in normal subjects and migraneurs. J Neurol.

[17] Fiermonte G, Annulli A, Pierelli F (1999). Transcranial doppler evaluation of cerebral hemodynamics in migraineurs during prophylactic treatment with flunarizine. Cephalalgia.

[18] Wallasch T-M (1992). Transcranial Doppler ultrasonic features in chronic tension-type headache. Cephalalgia.

[19] Wallasch T-M, Beckmann P, Kropp P (2011). Cerebrovascular reactivity during the Valsalva maneuver in migraine, tension-type headache and medication overuse headache. Funct Neurol.

[20] Savrun FK, Goksan B, Savrun M, Sahin R, Sahin S (2008). Cerebral blood flow changes in patients with probable medication-overuse headache. Funct Neurol.

[21] Lyngberg AC, Rasmussen BK, Jørgensen T, Jensen R (2005). Prognosis of migraine and tension-type headache: a population-based follow-up study. Neurology.

[22] Lieba-Samal D, Bartl S, Salhofer S, Prajsnar A, Massl R, Freydl E, Fathinia P, Wöber-Bingöl C, Wöber C (2009). The course of migraine-a diary study in unselected patients. Cephalalgia.

[23] Bigal ME, Lipton RB (2008). Clinical course in migraine: conceptualizing migraine transformation. Neurology.

